# Use of high-plex data provides novel insights into the temporal artery processes of giant cell arteritis

**DOI:** 10.3389/fimmu.2023.1237986

**Published:** 2023-09-06

**Authors:** Simon Parreau, Elsa Molina, Stéphanie Dumonteil, Radjiv Goulabchand, Thomas Naves, Melanie C. Bois, Hussein Akil, Faraj Terro, Anne-Laure Fauchais, Eric Liozon, Marie-Odile Jauberteau, Cornelia M. Weyand, Kim-Heang Ly

**Affiliations:** ^1^ Division of Rheumatology, Mayo Clinic, Rochester, MN, United States; ^2^ Division of Internal Medicine, Dupuytren University Hospital, Limoges, France; ^3^ INSERM U1308, Faculty of Medicine, University of Limoges, Limoges, France; ^4^ Stem Cell Genomics Core, Stem Cell Program, University of California, San Diego, La Jolla, CA, United States; ^5^ Next Generation Sequencing Core, Salk Institute for Biological Studies, La Jolla, CA, United States; ^6^ Division of Internal Medicine, Nîmes University Hospital, University of Montpellier, Nîmes, France; ^7^ Division of Gastroenterology, Department of Medicine, University of California, San Diego, San Diego, CA, United States; ^8^ Division of Laboratory Medicine and Pathology, Mayo Clinic, Rochester, MN, United States; ^9^ Cell Biology, Dupuytren University Hospital, Limoges, France

**Keywords:** giant cell arteritis, vasculitis, transcriptomic analysis, temporal artery biopsy, gene expression

## Abstract

**Objective:**

To identify the key coding genes underlying the biomarkers and pathways associated with giant cell arteritis (GCA), we performed an *in situ* spatial profiling of molecules involved in the temporal arteries of GCA patients and controls. Furthermore, we performed pharmacogenomic network analysis to identify potential treatment targets.

**Methods:**

Using human formalin-fixed paraffin-embedded temporal artery biopsy samples (GCA, n = 9; controls, n = 7), we performed a whole transcriptome analysis using the NanoString GeoMx Digital Spatial Profiler. In total, 59 regions of interest were selected in the intima, media, adventitia, and perivascular adipose tissue (PVAT). Differentially expressed genes (DEGs) (fold-change > 2 or < −2, p-adjusted < 0.01) were compared across each layer to build a spatial and pharmacogenomic network and to explore the pathophysiological mechanisms of GCA.

**Results:**

Most of the transcriptome (12,076 genes) was upregulated in GCA arteries, compared to control arteries. Among the screened genes, 282, 227, 40, and 5 DEGs were identified in the intima, media, adventitia, and PVAT, respectively. Genes involved in the immune process and vascular remodeling were upregulated within GCA temporal arteries but differed across the arterial layers. The immune-related functions and vascular remodeling were limited to the intima and media.

**Conclusion:**

This study is the first to perform an *in situ* spatial profiling characterization of the molecules involved in GCA. The pharmacogenomic network analysis identified potential target genes for approved and novel immunotherapies.

## Introduction

Giant cell arteritis (GCA), also called temporal arteritis or Horton’s disease, is the most common systemic large-vessel vasculitis; it primarily affects the aorta and its major branches in individuals aged over the age of 50s (1). This inflammatory disease is related to immune system aging (2) and a high risk of arterial occlusion, which can lead to irreversible blindness, strokes, and death (3, 4). Despite advances in understanding the underlying disease mechanisms of GCA over the past three decades, the treatment options are still limited. Furthermore, a high proportion of patients relapse during treatment with corticosteroids, methotrexate, or tocilizumab, which are the current recommended treatments for this vasculitis (5–8). In almost half of the patients, arterial inflammation persists even 1 year after corticosteroid treatment (9).

The management of GCA is challenging owing to its histopathological complexity, which occurs because it involves three arterial layers, from outer to inner: adventitia, media, and intima. Despite the potential significance of perivascular adipose tissue (PVAT) in the development of GCA, its role has not been thoroughly investigated (10). The disease process begins in the outer layer of the vessel, the adventitia, where resident dendritic cells are stimulated by an unknown trigger. This initiates an inflammatory response that involves infiltration by lymphocytes, macrophages, and giant cells, which damage the elastic tissue through release of metalloproteases and reactive oxygen species. As a result, vascular smooth muscle cells differentiate into myofibroblasts, migrate, and eventually cause intimal hyperplasia, leading to vessel obstruction (11).

Although the characterization of GCA through immunoprofiling of arterial tissue sections is essential for understanding immune cell interactions and evaluating the effects of immunosuppressive and anti-inflammatory drugs, previous studies had some limitations such as tissue heterogeneity or small sample sizes (12–15). Multiple studies have analyzed the transcriptomic profile of the entire artery without considering the anatomical characteristics of GCA lesions. As a result, transcriptional events specific to a particular arterial layer may have been missed when the entire artery was analyzed. Therefore, we used NanoString GeoMx^®^ Digital Spatial Profiler (DSP) technology to explore the molecular events involved in inflammatory and vascular remodeling in each sub-layer of GCA (16). Thanks to this technology that allows complex RNA profiling and quantification of the transcripts in specific regions within a tissue, we were able to study spatially the transcriptome of GCA and non-GCA areas of human temporal arteries using formalin-fixed, paraffin-embedded tissues. Whole tissue spatial transcriptomic analyses can better elucidate functional mechanisms and gene pathways in pathological tissues with complex organization, such as human temporal artery, which is the main artery involved in patients with GCA.

The present study is the first to provide *in situ* spatial transcriptomic analyses for the identification of layer-specific genes and cellular functions related to GCA, potentially leading to the identification of novel therapeutic targets. Here, we proposed a model in which spatiotemporal transcriptomic analysis can be used to identify previously unknown pathways and new drug targets for GCA. Therefore, our results can be useful for drug development strategies and may improve patient outcomes.

## Patients and methods

### Study design and participants

In this retrospective observational study, we recruited non-consecutive patients referred to the internal medicine department of the Limoges University Hospital Dupuytren, France, between January 1, 2014, and December 31, 2018. The participants underwent temporal artery biopsy (TAB) for suspected GCA, which was diagnosed based on the 2022 ACR/EULAR criteria (17) and histologically confirmed based on the presence of panarteritis with an inflammatory infiltrate (e.g., macrophages and lymphocytes) and fragmentation of the internal elastic lamella, corresponding to the most common histological pattern described by Hernández-Rodríguez et al. (18, 19). This study included patients who were treated with glucocorticoids for 1–10 days before TAB as this treatment does not affect the histopathological findings of TAB (20, 21). Participants with a negative TAB and a final diagnosis other than GCA were considered controls. We recorded the age, sex, symptoms, treatments, and outcomes of each participant at the time of diagnosis.

### Tissue collection and preparation

Unilateral TABs were performed under local anesthesia by an ophthalmologic surgeon using standard techniques (22). A 1–4-cm-long specimen of the temporal artery was obtained for histological analysis. The temporal artery specimens were analyzed using standard protocols. The specimens were subjected to gross evaluation and were cross sectioned serially. Tissues were fixed in 10% neutral buffered formalin for 24 h, followed by transfer to 70% ethanol before paraffin embedding. For each patient and control, the most representative segments were grouped into formalin-fixed paraffin-embedded (FFPE) temporal artery blocks using the tissue microarray method. Tissue microarrays were constructed from 2-mm-diameter cores punched from each FFPE tissue block.

### NanoString GeoMx Digital Spatial Profiler

The NanoString GeoMx DSP platform quantifies RNA abundance by counting unique indexing oligonucleotides assigned to each target of interest. Those oligonucleotides are covalently attached to mRNA hybridization probes with an ultraviolet-photocleavable linker (16). First, FFPE temporal artery blocks from GCA and control patients were sectioned to a thickness of 5 μm. The sections were mounted on an adhesive or positively charged slides (cat# S21.2113.A; Bond Plus slides; Leica Biosystems, Richmond, VA, USA) with a maximum of 24 individual tissues per slide at the San Diego Pathology Department (San Diego, CA, USA). Some slides were stored at 4°C, while others were deparaffinized and used for hematoxylin & eosin (H&E) staining (San Diego Pathology Department) for subsequent microscopy imaging (Keyence BZ-X800). A cardiovascular pathologist (M.C.B.) and two GCA specialists (S.P. and C.M.W.) reviewed all H&E-stained tissues. Overall, one GCA patient was classified as healed and excluded from this study because the artery exhibited no inflammatory infiltrate, and one control sample was also excluded because it showed tertiary lymphoid structure in the adventitia.

The freshly cut sections were stained with fluorescently labeled imaging reagents and mRNA markers as described previously (16). Briefly, sections were labeled with the cell markers, α-smooth muscle actin, CD3, and CD68, and the nuclear stain SYTO 13. The NanoString commercial Human Whole Transcriptome Atlas (GeoMx WTA) panel (18,000 protein-coding genes) was selected. Four slides (two with GCA patients and two with controls) were scanned on a GeoMx DSP instrument (NanoString Technologies Inc., Seattle, WA, USA) and individual regions of interest (ROIs) with a maximum diameter of 400 μm were created within each of the four arterial layers in GCA and controls.

In this study, we considered a sample as a control where no inflammatory infiltrate was observed, and the 4 artery layers (intima, media, adventitia, and PVAT) were distinguished on the basis of H&E staining as well as internal elastic lamina between intima and media; concentric smooth muscle cells in the media unlike the adventitia, and, finally, the presence of adipocytes in the PVAT unlike the adventitia. A representative portion of each layer was selected by two GCA specialists (S.P. and R.G.) and confirmed by a pathologist (M.C.B.) (**Supplemental Figure 1**). Once each ROI was compartmentalized, ultraviolet-cleaved indexing oligonucleotides were collected into a 96-well plate. Libraries were prepared according to the manufacturer’s instructions (protocol 01/2019). The library quality and quantity were assessed using the Bioanalyzer DNA High Sensitivity Analysis. Samples were sequenced on the NovaSeq 6000 platform (Illumina, San Diego, CA, USA) and reads were digitally quantified and normalized using GeoMx DSP Data Analysis software (Merritt).

### Data processing and analysis

Data obtained from the NanoString GeoMx DSP platform were analyzed using a dedicated software (GeoMx Analysis Suite 2.3). The quality control analysis showed that all ROIs had raw read counts above 1 million and good alignment rate and sequencing saturation. The local outliers were negative control probes and were removed from the ROIs. ROIs with deduplicated probe counts Q3 (upper quartile) < 2 were excluded. Furthermore, ROIs were removed if < 1% of their genes had read counts higher than the limit of quantification (LOQ). LOQ was defined as geomean (NegProbe) × geoSD(NegProbe)² for each ROI. Target filtering was applied to retain gene targets with read counts above LOQ in at least 10% of ROIs. Q3 normalization was applied on the filtered ROIs and gene targets.

The raw data were deposited at the NCBI Gene Expression Omnibus (GEO) database (https://www.ncbi.nlm.nih.gov/geo/query/acc.cgi?acc=GSE237441) under the accession number GSE237441.

Differential gene expression across groups was analyzed using linear mixed effect models; differentially expressed genes (DEGs) were defined as fold-change > 2 or < −2, and Benjamini-Hochberg-adjusted p < 0.01. Analyses were conducted using R software (version 3.2.2; R Foundation for Statistical Computing, Vienna, Austria).

The upregulated and downregulated DEGs were analyzed using DAVID Bioinformatics Resources software (version 2021; https://david.ncifcrf.gov/) for functional annotations and gene set enrichment analysis. DAVID was used to obtain statistically enriched Gene Ontology (GO) terms for biological processes (GOTERM_BP_FAT). GO terms with EASE score < 0.05 were considered statistically enriched. Based on the known mechanisms in GCA, pathways of interest, signaling cascades, and molecules were selected from among the dysregulated GO terms. These selections are presented as dot plots using ggplot2 package of R software (version 3.2.2).

Dysregulated genes per layer with a fold-change > 2 or < −2 were organized according to the STRING network (https://string-db.org/). The one or two main networks were represented for each layer. Genes disconnected from the main network were removed. An interaction score with the highest confidence (0.900) was used. The interaction sources excluded text mining. Using networks generated by the STRING network, a pharmacogenomic network was constructed by targeting existing druggable genes (https://www.dgidb.org/). Drugs with Food and Drug Administration (FDA) approval were selected.

### Fluorescence immunohistochemistry

From block, 4-μm-thick slices were cut and dewaxed by successive immersion in pure xylene (Sigma). Rehydration and unmasking of antigens were carried out by sequentially immersion into 100%, 90%, 80% and 70% ethanol baths (Sigma), phosphate-buffered saline (PBS; Gibco), and citrate buffer (pH 7). Saturation of nonspecific antigenic sites was achieved by incubation in PBS containing 3% bovine serum albumin (BSA). The primary antibodies were diluted in 3% BSA and incubated overnight at 4°C. The antibodies used were as follows: anti-CD74 (1:50, mouse, Abcam), anti-CD90 (1:50, rabbit, Abcam), anti-CD3 (dilution recommended by the supplier, rabbit, Ventana). The sections were subsequently incubated with a secondary antibody coupled to Alexa Fluor 594 or 488 (Molecular Probes) for 60 min. After washing with PBS, nuclear counterstaining was carried out with 4′,6-diamidino- 2-phenylindole (Sigma) and the sections were mounted in an aqueous medium (Vectashield, Sigma). Images were acquired with Leica microscope (DMi8, Leica).

### Role of the funding source

The funders of the study had no role in study design, data collection, analysis, or interpretation, or in writing the manuscript.

### Ethics

The study protocol (87RI21_0065) was approved by the Ethics Committee of the University Hospital of Limoges, France (23). Temporal artery tissues were obtained from the biological collection DC-2010-1079. All participants provided informed consent.

## Results

### Participant characteristics

The characteristics of GCA patients (n = 9) and controls (n = 7) are presented in the **Supplemental Table 1**. Among the controls, the final diagnoses were non-arteritic ischemic visual impairment (n = 4), peripheral seronegative polyarthritis (no argument for polymyalgia rheumatica) (n = 2), and infection-related encephalopathy (n = 1). Three controls had an inflammatory syndrome related to an acute infection. Among the entire cohort, one patient had diabetes (control), one had a previous cardiovascular complication (control), and two patients were active smokers (one control and one GCA). Corticoids were used before TAB for 8/9 GCA patients (mean 4.3 days before TAB, mean dose of 0.85 mg/kg/d) and 3/7 controls (mean 18.3 days before TAB, mean dose of 0.7 mg/kg/day).

**Figure 1:**
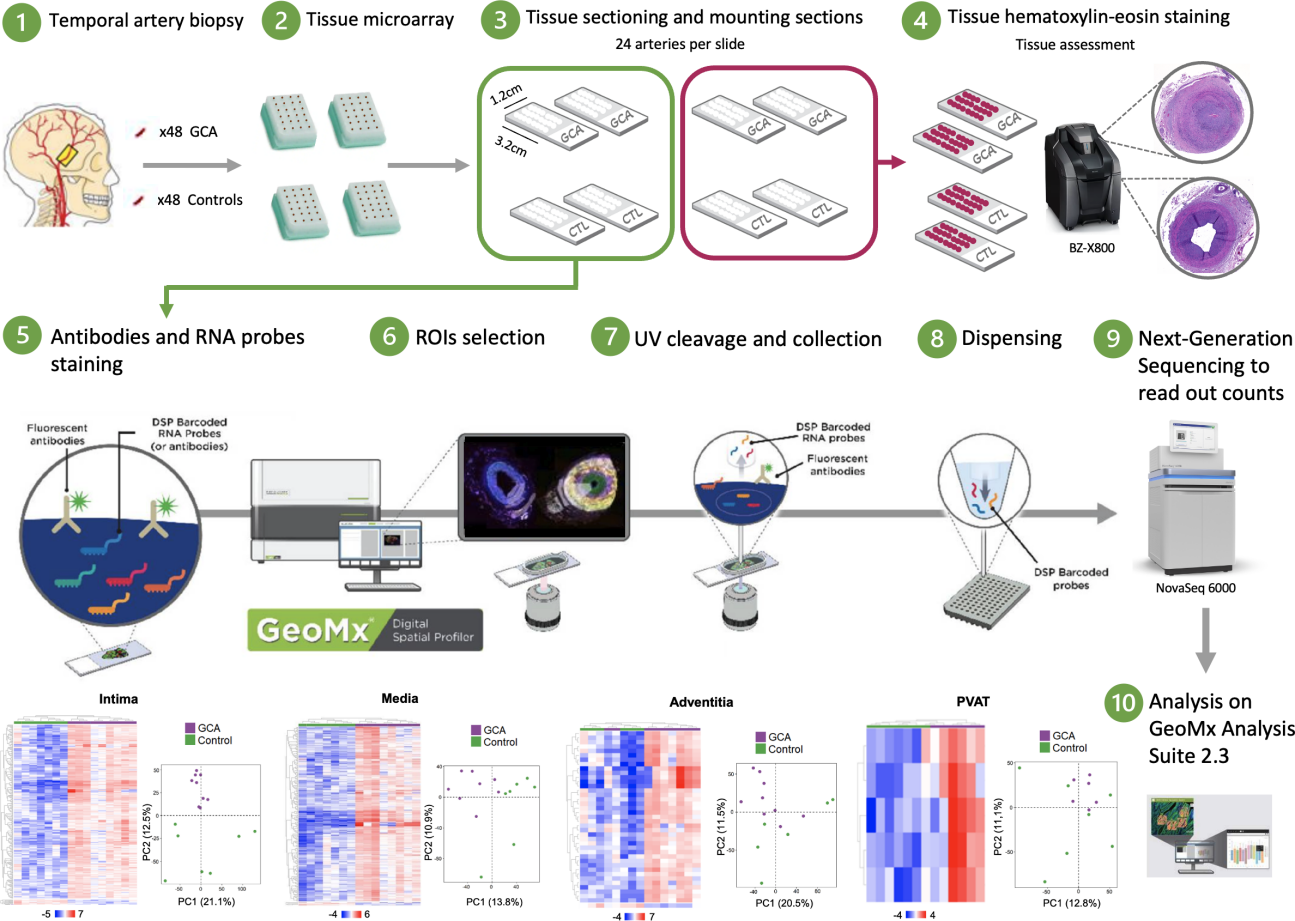
Schematic summary of the protocol study. Tissue microarrays (TMA) are prepared from a cohort of human temporal artery biopsies. Freshly cut sections of TMA are run through NanoString GeoMx DSP instrument. Synthetic DNA oligonucleotide barcodes are attached to *in situ* probes for mRNA detection via a UV photocleavable linker. A single tissue section is labeled with a cocktail of these probes and fluorescently labeled morphology markers. Regions of interest (ROI) for molecular profiling are selected based on the fluorescent images, and UV light illuminates each region independently, which releases the barcodes from that region for subsequent counting using Next-Generation sequencing.

All GCA patients had panarteritis involving the three arterial layers with destruction of the internal lamella on histological examination. The histological sections of the artery are presented in **Supplemental Figure 1**. Then temporal arteries from the 16 participants were subjected to transcriptional analysis for each arterial layer (intima, media, and adventitia) and the PVAT following technical pipeline indicated in **Figure 1**. Because the control arteries did not present CD3 and CD68 staining, layers were selected under the control of a senior pathologist depending on their macro characteristics. Four ROIs involving the media, adventitia, and PVAT were excluded from analysis due to poor quality, respectively. Altogether, we analyzed 59 ROIs, checked for quality and correspondence to each layer, from 16 participants to conduct our spatial transcriptomic study.

### Temporal artery whole transcriptome analysis exhibits different profiles depending on layers in giant cell arteritis

**Figure 2:**
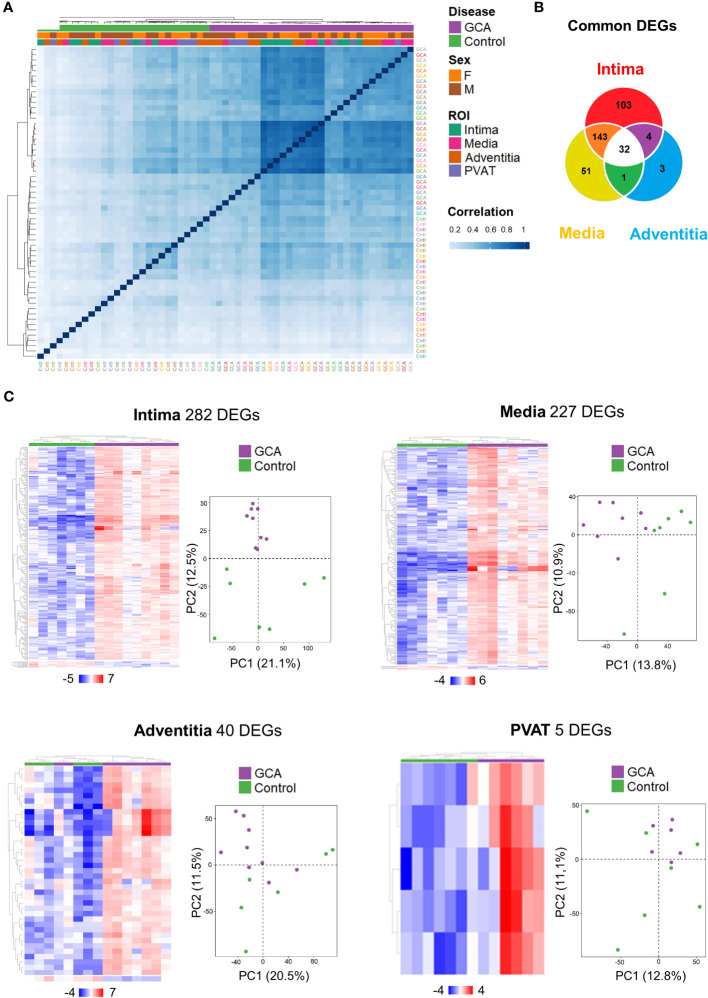
Temporal Artery Whole Transcriptome Analysis across layers. **(A)** Hierarchical clustering according to the correlation between the 59 ROIs studied with identification of status (Giant Cell Arteritis or Control), gender (Male or Female) and layers (Intima, Media, Adventitia, Perivascular adipose tissue). **(B)** A total of 337 differentially expressed genes (DEGs) were identified for the intima, media, and/or adventitia of GCA compared with controls (fold change < -2 or > 2, adjusted p-value < 0.01). Some genes are only dysregulated in one layer and others are shared by two or three arterial layers. **(C)** Hierarchical clustering of patients by DEGs and principal component analysis on gene expression profiles (12,076 genes in total) from temporal artery biopsy samples in both patient groups (Giant Cell Arteritis and control temporal arteries) for each different layer. GCA, giant cell arteritis; F, female; M, male; PVAT, perivascular adipose tissue; DEGs, differential expressed genes; PC, principal component.

As attempted, correlation analysis demonstrated that disease status (GCA and control) only differentiated samples (**Figure 2A**). Interestingly, Pearson correlation coefficient showed that GCA transcriptome remains more homogeneous than in controls as illustrated by increasing intensity of blue reflecting correlation index tending toward 1.

A total of 12,076 genes were detected and 337 different genes (2.8%) were dysregulated between GCA and control participants (fold-change < −2 or > 2, p-adjusted < 0.01). Venn diagram presents unique and common DEGs for the intima, media, and adventitia (**Figure 2B**). The number of DEGs increased from external layer, adventitia with 40 genes, to media and intima with respectively 227 and 282 genes. In total, 32 common DEGs were significantly identified among the three layers (intima, media and adventitia), corresponding to the major histocompatibility complex (*CD74, B2M, HLA-DRB1, HLA-A, HLA-B, HLA-C*, and *HLA-DRA*), macrophages (*CD68*), immunoglobulins (*IGKC* and *IGHG1-4*), apolipoprotein (*APOC1*), cathepsins (*CTSB* and *CTSZ*), and tissue remodeling (*ADAM15, COL1A2, COL3A1, TMSB10*, and *ARHGDIB*). The other common genes were *NPIPB6, PABPC1*, and those associated with ribosomes (*RPL23, RPL28, RPL31, RPL37, RPS12, RPS28, RPS29, RPS3*, and *RPS4X*). Patterns for the four layers in each participant are shown in **Figure 2C**. Principal component analysis (PCA) highlighted that only intima present a distribution profile more homogeneous and reproducible than other GCA layers and control samples.

Altogether, these results highlight that the intima is the layer where the transcriptome is the most dysregulated depicting a gradient of decreased DEG from the internal to the periphery of the artery.

### Top differentially expressed genes across layers in giant cell arteritis compared to control temporal arteries

**Figure 3:**
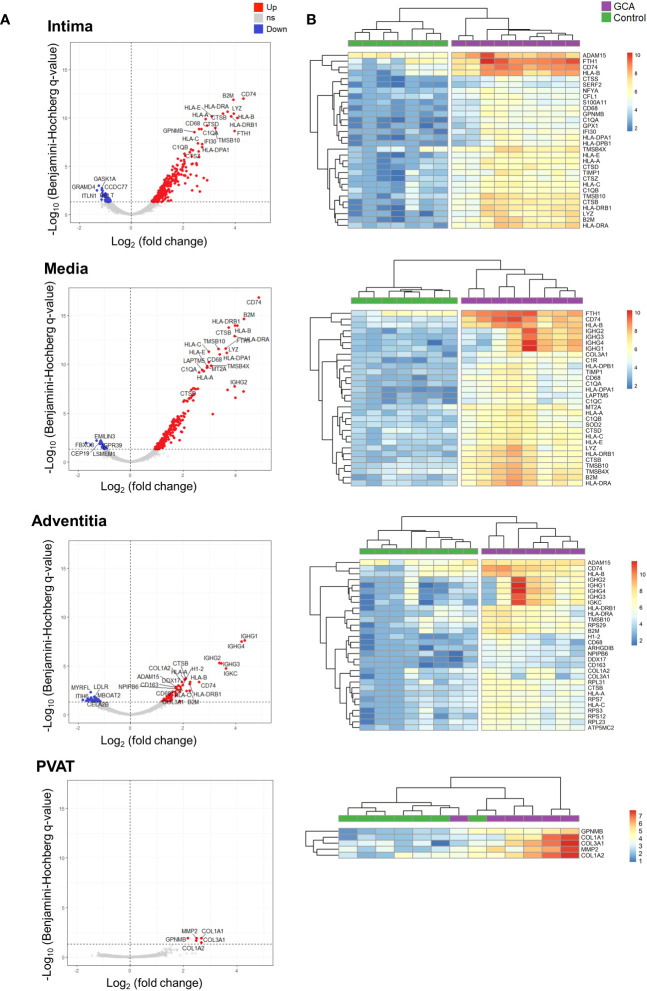
Top differentially expressed genes (DEGs) across layers in Giant Cell Arteritis compared to control temporal arteries. **(A)** Top 20 upregulated genes and top 5 downregulated genes for each layer (intima, media, adventitia, and perivascular adipose tissue) of Giant Cell Arteritis temporal arteries. **(B)** Hierarchical clustering among each ROI participant for the top 30 dysregulated genes per layer. Up, up-regulated genes; ns, non statistically significative dysregulated genes; Down, down-regulated genes; GCA, giant cell arteritis; PVAT, perivascular adipose tissue.

Overall, most DEGs were upregulated in ROIs from GCA compared to controls. The top 20 upregulated and top 5 downregulated genes per arterial layer in GCA patients compared to the controls are shown in **Figure 3A**. The highest numbers of DEGs were observed in the media and intima.

**Figure 3B** presents the top 30 dysregulated genes across arterial layers between both studied groups. In the media and intima, *CD74* was the most upregulated gene between GCA and control specimens. Healthy temporal arteries do not exhibit CD74 staining on immunofluorescence analysis (**Supplemental Figure 2**). In contrary, GCA arteries has CD74+ cells, particularly within the intima, close to the media border, as well as in the media and the adventitia. Also, cells expressing CD74 are also CD90+; however, they do not express CD3, marker for lymphocytes. Finally, migration inhibitory factor (MIF), a receptor for CD74, was significantly overexpressed in the intima of GCA compared to controls (p-adj = 0.0031).

Altogether, these results unveiled that most dysregulated genes in the three arterial layers belong to the antigen presentation gene family and vascular remodeling functions.

### Immune related functions and vascular remodeling pathways are predominant in media and intima

**Figure 4:**
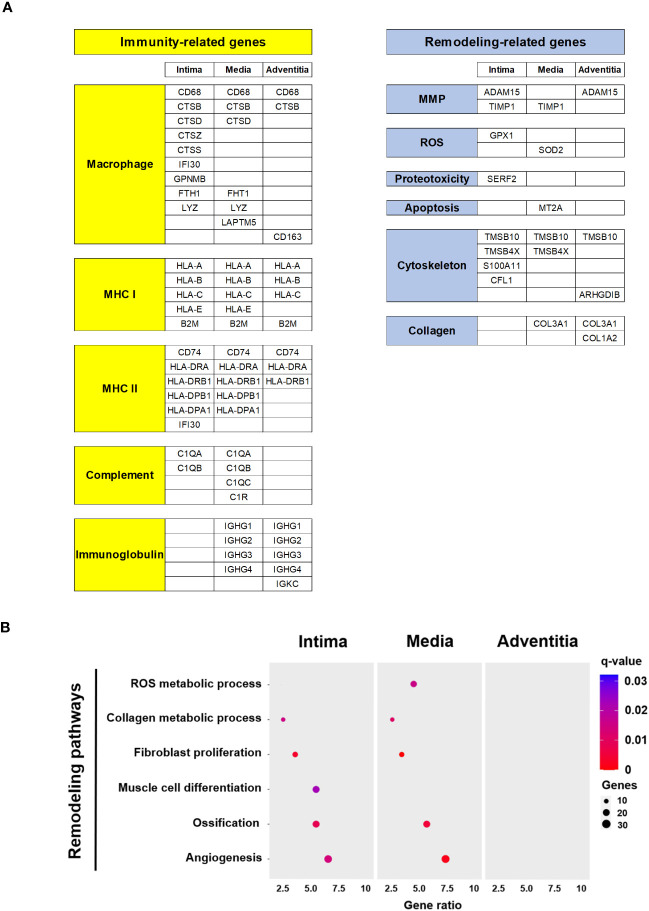
Functions and pathways related top DEG in Giant Cell Arteritis temporal arteries. **(A)** Top 30 DEG per layer (intima, media, adventitia) categorized by immune and remodeling functions **(B)** Dot plot showing significant remodeling related pathways according to layer (intima, media, adventitia). MHC, major histocompatibility complex; MMP, metalloproteinase; ROS, reactive oxygen species.

We classified the top 30 DEGs by arterial layers according to their main functions (**Figure 4A**). Most top DEGs per layer were associated with immune or remodeling functions. Immune function-related genes were associated with macrophages (*CD68* and cathepsins), major histocompatibility complex, complement system, and immunoglobulins. Remodeling-related genes were mainly associated with metalloproteases, reactive oxygen species, cytoskeleton organization, and collagen.

Using the DAVID tool, remodeling-related pathways of interest with gene dysregulation were selected (**Figure 4B**). PVAT was excluded due to the low number of dysregulated genes between GCA and control specimens. Remodeling pathways associated with GCA primarily involve the intima and media and include the reactive oxygen species and collagen metabolic process, fibroblast proliferation, muscle cell differentiation, ossification, and angiogenesis. The dysregulated genes for each signaling pathway are detailed in **Supplemental Figure 3**.

Altogether these results suggest that activated immune cells are actively and mainly present in the intima and the media layers. Likewise, vascular remodeling process following a gradient from the outside to inside part of the vessel.

### Meta-analysis studies similar expression profiles among genes of interest across arterial layers

**Figure 5:**
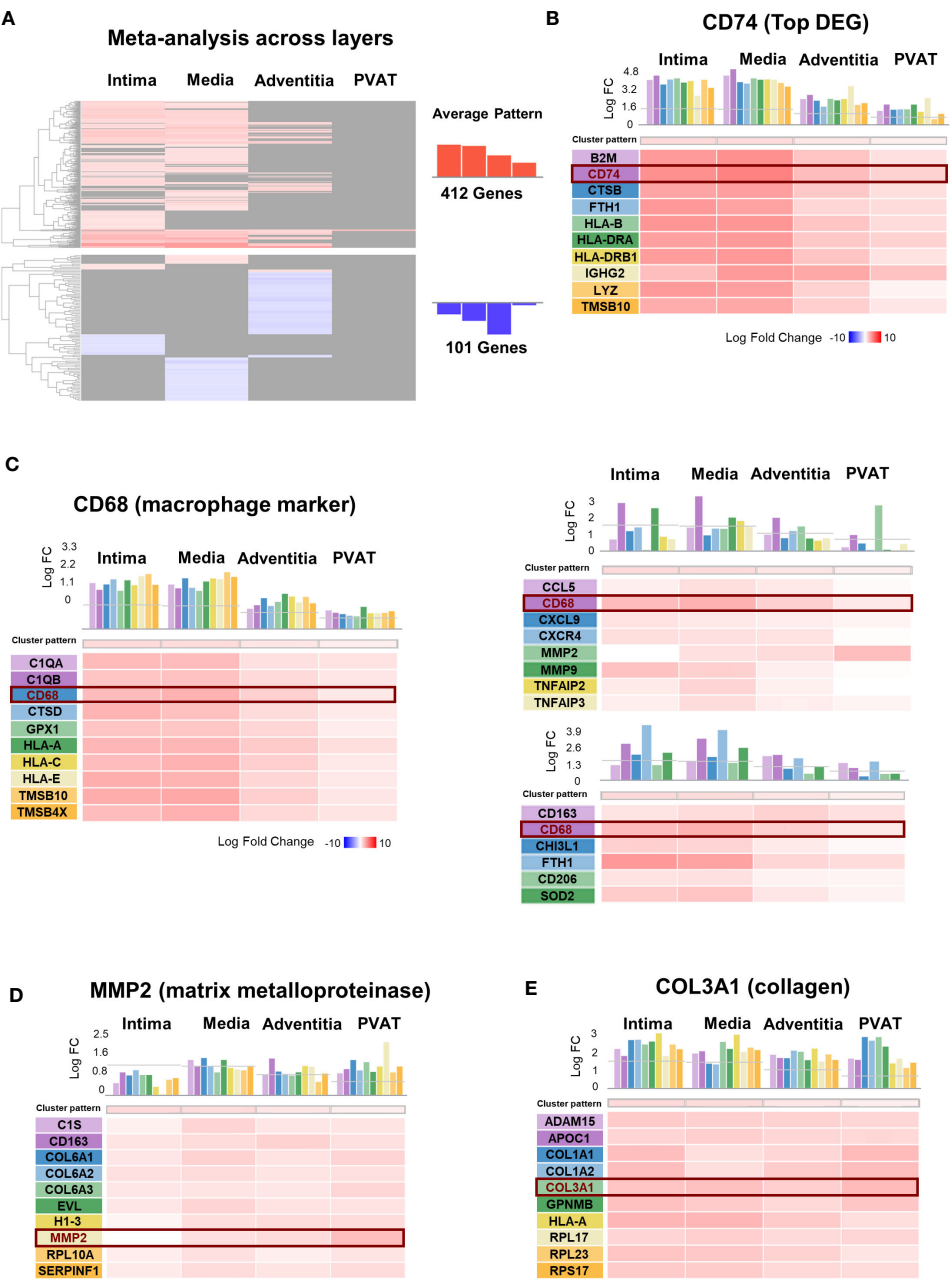
Meta-analysis across layers of Giant Cell Arteritis temporal arteries. **(A)** Hierarchical clustering of 513 dysregulated genes in giant cell arteritis (GCA) across layers. **(B–F)** Genes presenting the same expression profiles across layer for: **(B)** CD74, the most dysregulated gene in GCA compared to control; **(C)** CD68, macrophage marker; the two main genes dysregulated and implicated in vascular remodeling, **(D)** MMP2, a matrix metalloproteinase type 2 marker, **(E)** COL3A1, collagen type III alpha-1 marker. PVAT, perivascular adipose tissue; DEG, differential expressed gene; FC, fold-change.

The DEGs among the four layers were compared (**Figure 5A**). The meta-analysis identified 412 upregulated genes and 101 downregulated genes. Genes whose expression profiles were similar to *CD74*, the top DEG, are shown in **Figure 5B**. These genes were mainly involved in antigen presentation, including the major histocompatibility complex (*B2M, HLA-B, HLA-DRA*, and *HLA-DRB1*), cathepsin (*CTSB*), ferritin (*FTH1*), immunoglobulin (*IGHG2*), and lysozyme (*LYZ*).

As macrophages are the key immune cells in GCA, we presented the genes with a similar expression profile within the four layers like CD68, a marker for macrophages (**Figure 5C**). The DEGs included those related to cytokines (*TNF*), chemokines (*CCL5, CXCL9*, and *CXCR4*), YKL40 (*CHI3L1*), metalloproteases (*MMP2* and *MMP9*) secreted by macrophages, membrane receptors (*CD63* and *CD206*), and iron metabolism-related molecules (*FTH1* and *SOD2*). Similarly, we evaluated vascular remodeling during GCA based on the two mains associated DEGs: *MMP2* (**Figure 5D**) and collagen *COL3A1* (**Figure 5E**). We identified the top genes with the same expression profile across the layers.

### Potential novel drug-targeted pathways for GCA treatment

**Figure 6:**
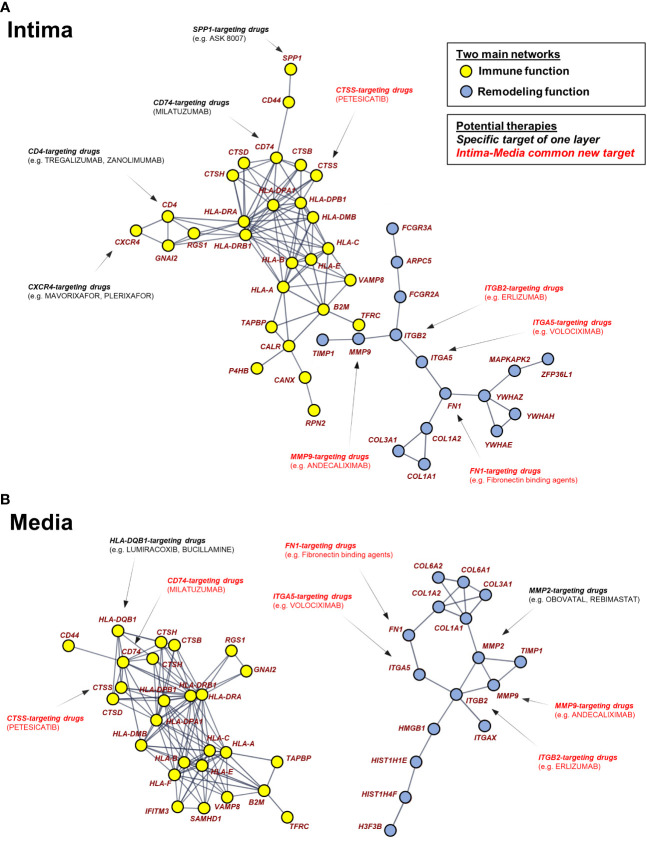
Pharmacogenomic network analysis uncovers drug-gene interaction landscape in Giant Cell Arteritis. The two largest connected components in the DEG-based protein-protein interaction network are composed of 43 up-regulated genes (nodes) in intima **(A)** and 42 up-regulated genes in media **(B)**. Using pharmacological information, genes were identified as druggable with FDA-approved, pharmaceutical drugs. Pharmaceutical drugs are divided into 2 types: specific target of one layer and intima-media common drugs. GCA, giant cell arteritis.

Transcriptomic profiling is an efficient and essential tool for drug target discovery. Several studies have combined gene expression profiling with systematic approaches for drug re-repurposing or biomarker discovery (24, 25). Among strategies for novel drug target identification, the network-based framework uses curated biological network topology to investigate the association across genes, diseases, and drugs (26). We constructed a pharmacogenomic network by integrating DEGs from each artery layer, a high-confidence protein-protein interaction network, and drug-gene interactions. Then, the top dysregulated genes are presented as a STRING analysis to highlight their relationships. The significantly connected genes are shown in **Figure 6**. Networks show the strong relationships between several genes. Two mains separate networks exist for each layer corresponding to global immune function (yellow) and vascular remodeling (blue). Key genes are present at nodes of those interactions and connected to other genes.

Approved therapies targeting these genes are presented, of which have not been evaluated in relation to GCA previously. The spatial approach highlights therapies targeting top DEGs common to the intima and the media (red). Therapies targeting a gene only present in one network layer are also represented (black).

## Discussion

In the present study, we performed a spatial transcriptome-wide expression analysis to identify key genes and biomolecular processes involved in temporal arteritis. By comparing gene expression patterns within the different layers of the temporal arteries among GCA and non-GCA patients, we saw a significant upregulation - rather than down-regulation - of genes in all arterial layers from GCA in comparison to controls. This could suggest some insufficient regulatory mechanisms occurring during GCA, and overriding of regulatory functions. Intima and media layers were particularly affected by gene expression dysregulation in GCA in comparison to the other layers, as well as compared to controls. Differences between gene expressions were less obvious in the adventitia, which may be due to the low number of inflammatory cells in the adventitia from the two TABs of GCA patients (GCA#4 and GCA#5). Furthermore, the initial mechanism of GCA involves adventitial damage and stimulation of resident dendritic cells via toll-like receptors by an unknown stimulus (27). As TABs are often performed at an advanced stage of GCA when vascular remodeling has already occurred, the adventitia may be less affected by dysregulation than the media and intima at this stage. The PVAT did not show major transcriptomic differences between the groups which might be due to the low number of cells present within the ROIs compared to the other layers. Although the perivascular layer has been poorly studied in GCA, it may be involved in the inflammation of the adjacent artery like it has been shown in other vascular diseases (28, 29). A previous study has suggested the involvement of the periadventitial tissue in a subgroup of GCA (30), however, in our study, all the GCA patients did not exhibit the histological pattern they described.

*CD74* was the most dysregulated gene across layers in GCA. This gene encodes a cell transmembrane receptor of the macrophage migration inhibitory factor (31), which is expressed on antigen presenting cells (dendritic cells, macrophages, and B lymphocytes) and non-immune cells (epithelial and endothelial cells). *CD74* is known to be involved in apoptosis, immune response, and cell migration. Notably, *CD74* plays a role in tissue repair and wound healing. For instance, *CD74* deficiency protects against glomerulonephritis in lupus (32). Interestingly, CD74 is a ligand for the macrophage migration inhibitory factor (MIF), which is over-expressed in the serum of patients with GCA (33).

In the present study, most genes with the same pattern as *CD74* expression across different layers were involved in the immune response and belonged to the major histocompatibility complex classes I (*B2M, HLA-A*, and *HLA-B*) and II (*HLA-DRA* and *HLA-DRB1*) (34).

Next, we focused on macrophages, as they are the main immune cells involved in GCA. Macrophages can form granulomas and fuse together to produce giant cells. Lymphocytes recruit and activate macrophages in arteries through secretion of cytokines and growth factors (IL-6, IL-17, TNF-α, IFN-γ, and GM-CSF). Two main populations of CD68+ macrophages have been identified in GCA: adventitial macrophages, which are involved in TGF-β1, IL-6, and IL-1β secretion, and intimal and medial macrophages, which are involved in reactive oxygen species, nitric oxide, and metalloproteases secretion (13). They can also produce PDGF and VEGF. Under the combined action of MMP, PDGF, and VEGF, the internal elastic lamella of the media is destroyed, and vascular remodeling occurs. In the present study, the main DEGs with the same expression pattern as *CD68* were related to antigen presentation. The *FTH1* and *SOD2* genes, related to M1 macrophage polarization, exhibit the same expression pattern as *CD68* among the arterial layers, as compared to *CD163, CD206*, and *CHI3L1*, which are linked to M2 macrophages (35). Previous studies have demonstrated heterogeneity among CD68 macrophages in GCA temporal arteries (36, 37). Interestingly, the expression profiles of *CTSB* and *TMSB10* are similar to the one of CD68, which is related to the promotion of M2 macrophages (38, 39). On the other hand, *FTH1* encodes the major intracellular iron storage protein (ferritin heavy chain 1) and is expressed abundantly by M1 macrophages (35). We note that lymphocytic activation was less marked than that of macrophages. As most of the GCA patients in our study had glucocorticoids before TAB, the Th17 population was probably reduced, which could explain a small lymphocyte activation as demonstrated by Deng et al. (40).

Vascular remodeling plays a key role in GCA. Intimal hyperplasia is the main cause of arterial occlusion and leads to often-irreversible ischemic complications, which depend on vascular muscle cell differentiation and fibroblast proliferation, as described previously (24). No significant differences were observed between GCA patients and controls in terms of the expressions of IL-1 and IL-6 genes, although these cytokines are produced in the temporal arteries (19). These findings suggest that vascular remodeling has already started when TAB was performed. In this study, dysregulated remodeling is mainly correlated with the intima and media layers. The metalloproteinase genes *MMP2* and *MMP9* were overexpressed, which was also shown in previous studies (41). The release of reactive oxygen species by macrophages leads to lipid peroxidation of the membrane phospholipids of vascular smooth muscle cells, resulting in their combination with MMP and apoptosis (42). Furthermore, PDGF secreted by macrophages and giant cells participate in intimal hyperplasia (43). Neoangiogenesis comes from the action of VEGF secreted by macrophages. Our study demonstrated that angiogenesis occurs in the intima and media as previously published (43). Interestingly, ossification and calcification have never been thoroughly studied in GCA. Here, we demonstrated that these processes are present within the intima and the media, and are likely to play a role in the pathophysiology of GCA (44). Moreover, ossification cluster data are consistent with the overexpression of COL1A1, MMP9 and COL2A1, which have been previously found in GCA (45–47).

Finally, we built a pharmacogenomic network based on DEGs and Food and Drug Administration (FDA) approved drugs targeting these genes. By providing a system-wide view of mechanistic gene interactions, our spatial pharmacogenomic network can facilitate the design of novel pharmacologic intervention strategies. Our results suggest that macrophages are a promising target in GCA. In particular, granulocyte-macrophage colony-stimulating factor can be blocked by mavrilimumab, a growth factor for macrophage maturation (48, 49). Our pharmacogenomic network shows the potential benefit of blocking several macrophage-related genes using *MMP2*/*MMP9*- and *CXCR4*-targeting drugs. *CD74* being, in this study, the top DEG common to the three arterial layers in GCA, this gene could be another potential drug target to consider. Interestingly, the anti-CD74 drug milatuzumab has been evaluated in hematological diseases with promising results so far (50).

The present study has several strengths. First, we used an innovative technique for human artery spatial analysis. Second, this is the first whole-transcriptome analysis of TABs in GCA using the NanoString GeoMx DSP. Third, we identified a large number of DEGs, even after multiple hypothesis correction, indicating that, despite the limited number of patients studied here, the gene expression differences between GCA and control temporal arteries are robust and reliable (fold-change < −2 or > 2 with p-adjusted value < 0.01). There were a few limitations to this study. First, the study had a relatively small sample size (9 GCA patients and 7 controls) compared to what it is commonly conducted in GCA research. Also, all GCA patients exhibited panarteritis but no other histological patterns were observed. Because of the sample size, clinical characteristics of patients (large-vessel vasculitis, cranial disease, relapse, or remission) could not be significantly analyzed. Some controls had an inflammatory background (2 peripheral seronegative arthritis and 1 infection related encephalopathy), but these 3 controls had a negative TAB with no inflammatory infiltrate. Finally, our study used FFPE samples from TAB specimens but did not analyze cells within circulation.

In conclusion, we uncovered the transcriptomic signatures of GCA arteries on a regional scale spatially according to the arterial layers for the first time using the GeoMx DSP. This novel technique allows to better understand GCA and identify new therapeutic targets. For that, we used a pharmacogenomic network-based approach to highlight the targeted genes and therapeutics for a potential multimodal treatment of GCA. We hope our findings will be useful for further whole-tissue and single-cell multiomics profiling studies investigating the biomolecular pathways, networks, and biomarkers in GCA.

## Data availability statement

The data presented in the study are deposited in the NCBI GEO repository, accession number GSE237441.

## Ethics statement

The studies involving humans were approved by Ethics Committee of the University Hospital of Limoges. The studies were conducted in accordance with the local legislation and institutional requirements. The participants provided their written informed consent to participate in this study.

## Author contributions

SP and EM designed this study. EL collected data on clinical and patient characteristics. SP, MB, and CW reviewed the temporal artery sections. SP and RG selected the ROIs. FT and HA performed immunostaining. SP, EM, RG, and TN wrote the manuscript. EL, A-LF, M-OJ, CW, and K-HL reviewed the manuscript. All authors contributed to the article and approved the submitted version.

## Supplementary material

Supplementary Figure 1 Sections of arteries with selected region of interest (intima, media, adventitia, perivascular adipose tissue) from each study participant: left hematoxylin eosin staining; right CD68 (yellow) CD4 (red) alpha-smooth muscle actin (green) SYTO 13 (blue) staining. GCA, giant cell arteritis; A, adventitia; M, media; I, intima; PVAT, perivascular adipose tissue.

Supplementary Figure 2 Immunofluorescence analysis by microscopy of healthy and GCA temporal arteries. **(A)**: In healthy artery, cells do not express CD74. **(B)**: In GCA arteries, CD90+ cells express CD74, particularly in the outer part of the neointima near the media. CD3+ cells do not express CD74. GCA, giant cell arteritis, A, adventitia, M, media, NI, neointima.

Supplementary Figure 3 Dysregulated genes associated with remodeling pathways across layers in Giant Cell Arteritis compared to control temporal arteries. GCA, giant cell arteritis. The English in this document has been checked by at least two professional editors, both native speakers of English. For a certificate, please see: http://www.textcheck.com/certificate/xvD6Vy.
