# Supplementary figures and images for: Use of high-plex data provides novel insights into the temporal artery processes of giant cell arteritis
Source: Front Immunol. 2023 Sep 6;14:1237986. doi: 10.3389/fimmu.2023.1237986 (PMC10512077; doi:10.3389/fimmu.2023.1237986)

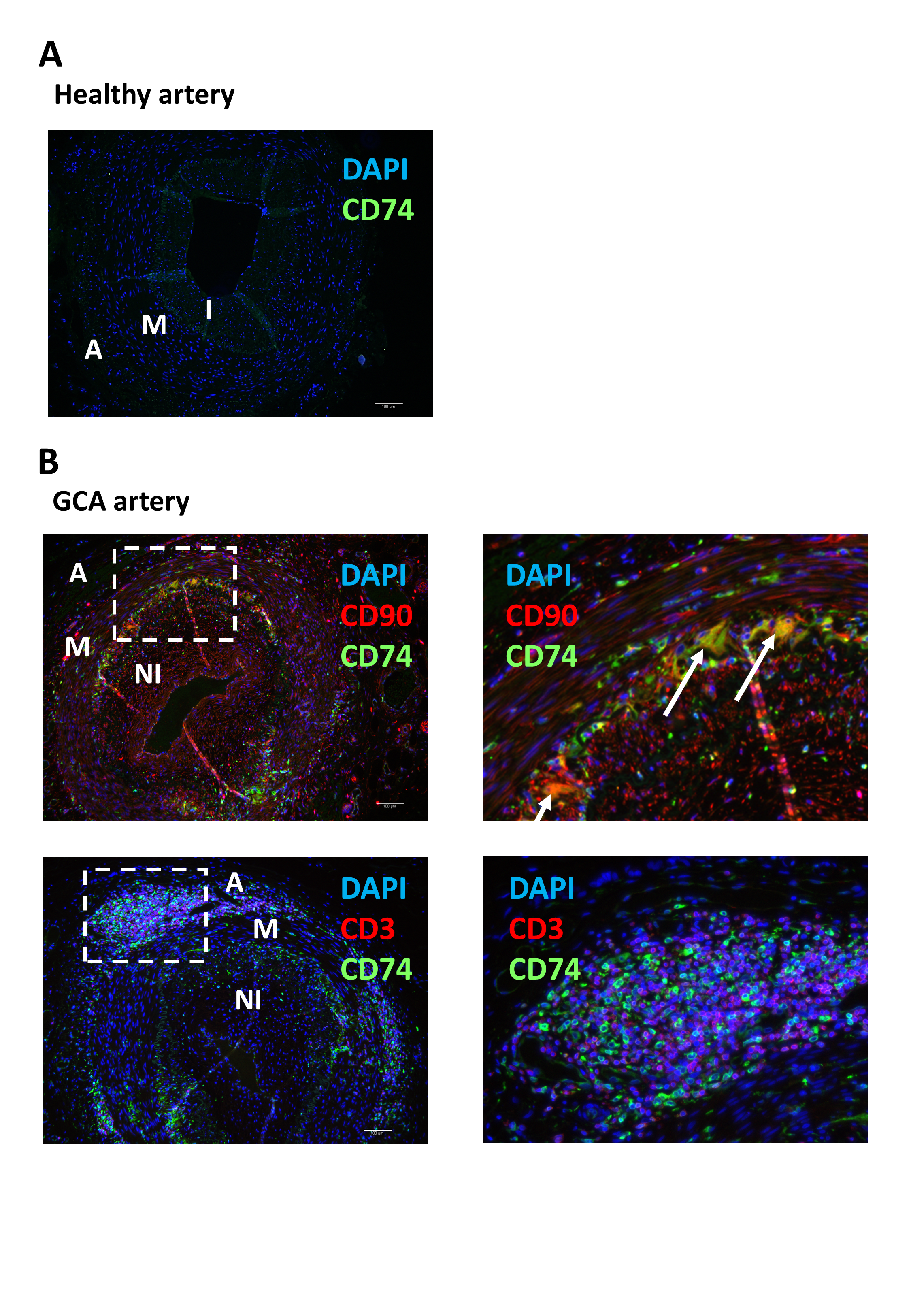

Supplement: Supplementary Figure 1 — Sections of arteries with selected region of interest (intima, media, adventitia, perivascular adipose tissue) from each study participant: left hematoxylin eosin staining; right CD68 (yellow) CD4 (red) alpha-smooth muscle actin (green) SYTO 13 (blue) staining. GCA, giant cell arteritis; A, adventitia; M, media; I, intima; PVAT, perivascular adipose tissue. [file Image_1.tif]

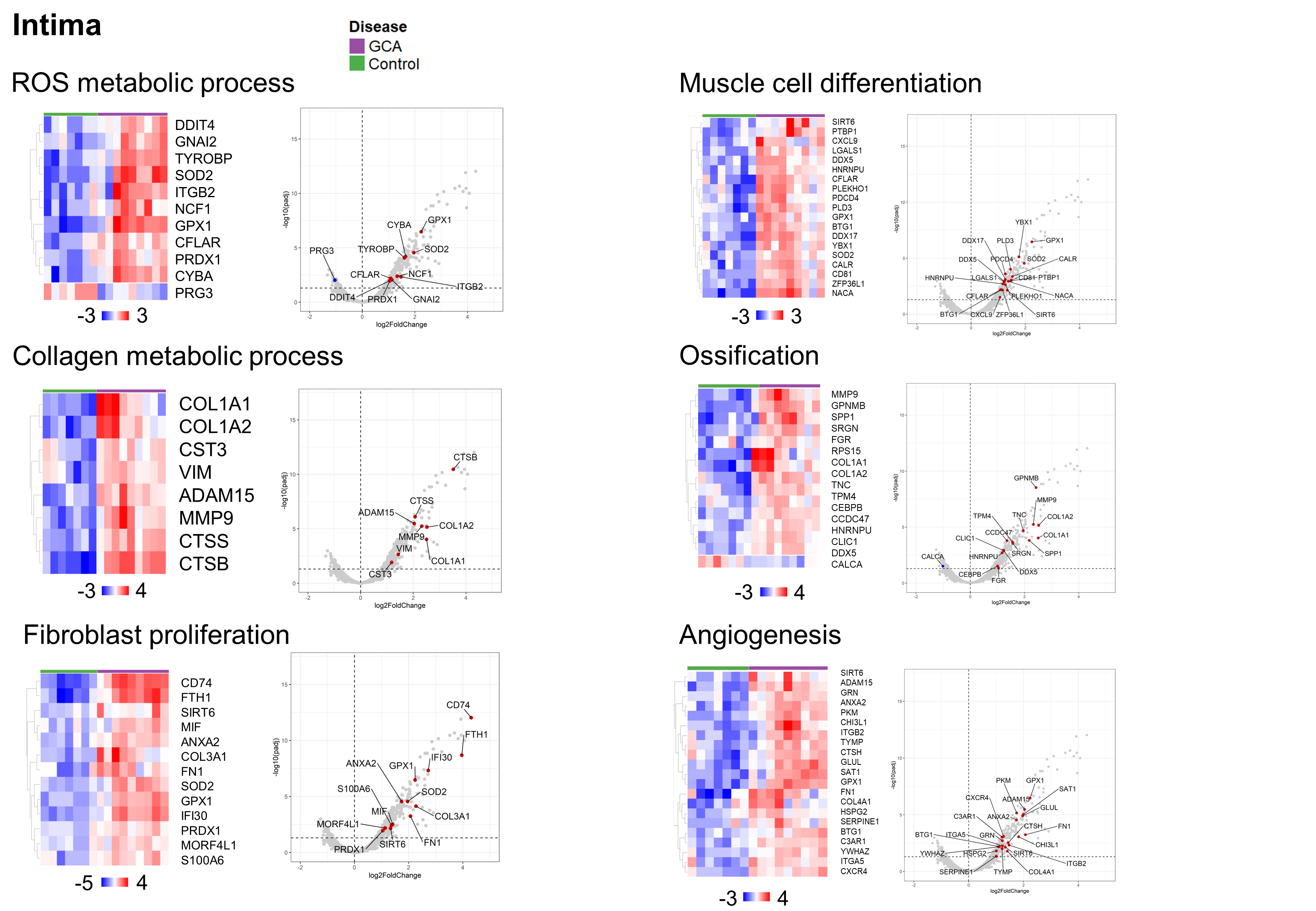

Supplement: Supplementary Figure 2 — Immunofluorescence analysis by microscopy of healthy and GCA temporal arteries. (A): In healthy artery, cells do not express CD74. (B): In GCA arteries, CD90+ cells express CD74, particularly in the outer part of the neointima near the media. CD3+ cells do not express CD74. GCA, giant cell arteritis, A, adventitia, M, media, NI, neointima. [file Image_2.tif]

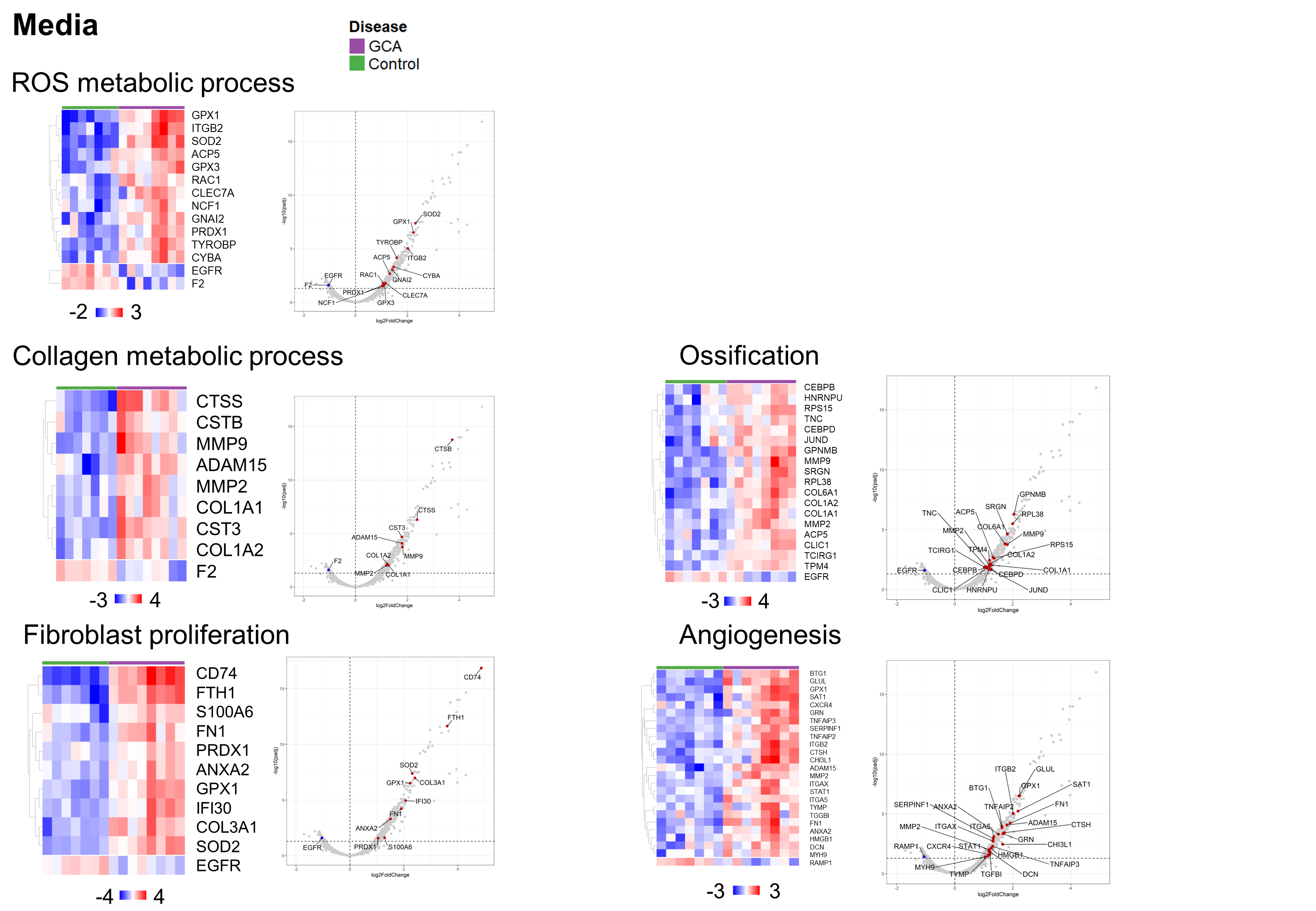

Supplement: Supplementary Figure 3 — Dysregulated genes associated with remodeling pathways across layers in Giant Cell Arteritis compared to control temporal arteries. GCA, giant cell arteritis. The English in this document has been checked by at least two professional editors, both native speakers of English. For a certificate, please see: http://www.textcheck.com/certificate/xvD6Vy. [file Image_3.tif]
